# Radioactive gold nanoparticles coated with BSA: A promising approach for prostate cancer treatment

**DOI:** 10.7150/ntno.91507

**Published:** 2024-01-01

**Authors:** Angélica Bueno Barbezan, Wilmmer Alexander Arcos Rosero, Daniel Perez Vieira, Maria Eduarda Zaganin Rigo, Giovana Dias da Silva, Alex Alves Rodrigues, Luís Fernando de Almeida, Fábio Fernando Alves da Silva, Andy González Rivera, Natanael Gomes da Silva, Emerson S. Bernardes, Carlos Alberto Zeituni, Maria Elisa C. M. Rostelato

**Affiliations:** 1 IPEN /CETER (Instituto de Pesquisas Energéticas e Nucleares / Centro de Tecnologia das Radiações), Brazil.; 2 IPEN /CECRF (Instituto de Pesquisas Energéticas e Nucleares / Centro de Radiofármacia), Brazil.; 3 IPEN / CBIO (Instituto de Pesquisas Energéticas e Nucleares / Centro de Biotecnologia), Brazil.

**Keywords:** Radioactive Gold Nanoparticles (AuNPs), Nanobrachytherapy, Prostate Cancer, Cytotoxicity, Therapeutic Efficacy

## Abstract

**Background:** Nanotechnology has revolutionized medicine, especially in oncological treatments. Gold nanoparticles (AuNPs) stand out as an innovative alternative due to their biocompatibility, potential for surface modification, and effectiveness in radiotherapeutic techniques. Given that prostate cancer ranks as one of the leading malignancies among men, there's a pressing need to investigate new therapeutic approaches.

**Methods:** AuNPs coated with bovine serum albumin (BSA) were synthesized and their cytotoxicity was assessed against prostate tumor cell lines (LNCaP and PC-3), healthy prostate cells (RWPE-1), and endothelial control cells (HUVEC) using the MTS/PMS assay. For *in vivo* studies, BALB/C Nude mice were employed to gauge the therapeutic efficacy, biodistribution, and hematological implications post-treatment with BSA-coated AuNPs.

**Results:** The BSA-coated AuNPs exhibited cytotoxic potential against PC-3 and LNCaP lines, while interactions with RWPE-1 and HUVEC remain subjects for further scrutiny. Within animal models, a diverse therapeutic response was observed, with certain instances indicating complete tumor regression. Biodistribution data emphasized the nanoparticles' affinity towards particular organs, and the majority of hematological indicators aligned with normative standards.

**Conclusions:** BSA-coated AuNPs manifest substantial promise as therapeutic tools in treating prostate cancer. The present research not only accentuates the nanoparticles' efficacy but also stresses the imperative of optimization to ascertain both selectivity and safety. Such findings illuminate a promising trajectory for avant-garde therapeutic modalities, holding substantial implications for public health advancements.

## Introduction

Nanotechnology, characterized by the design and manipulation of structures at the nanometric scale (1-100 nm), has catalyzed significant advancements across various disciplines, most notably in medicine 1. One emerging application is nanobrachytherapy, which involves the use of radioactive nanoparticles for targeted treatment of malignant neoplasms 2. Radiotherapy is a therapeutic modality used primarily in the treatment of cancer, using ionizing radiation to destroy or inhibit the growth of tumor cells. Within radiotherapy, brachytherapy is a specific technique where sealed radioactive sources are placed directly in or near the target tissue, allowing for the delivery of high doses of radiation to the tumor while minimizing exposure to surrounding healthy tissues. Recently, nanobrachytherapy has emerged as an innovation in this field, incorporating radioactive nanoparticles that provide a more precise and homogeneous radiation distribution, enhancing tumor treatment and further reducing side effects in adjacent healthy tissues. Gold nanoparticles (AuNPs) are garnering attention due to their unique properties: biocompatibility, high atomic number (benefiting radiotherapeutic techniques), and capability for surface modification for specific purposes 3.

In the 19th century, Michael Faraday was a pioneer in documenting the synthesis of gold nanoparticles (AuNPs) using phosphorus-based reducing agents in a scientific paper, highlighting their unique characteristics. Since this discovery, the field of nanoparticles has gained momentum, resulting in numerous publications exploring variations in size, shape, and functionalization of AuNPs. The preparation of AuNPs can be achieved through chemical, physical, and electrochemical techniques, with chemical methods traditionally favored for their simplicity and homogeneous results. However, the prevalence of potentially harmful reagents in such processes has driven the search for more sustainable and environmentally friendly approaches 4.

The literature is abundant with studies on the synthesis of gold nanoparticles, covering a variety of shapes such as nanospheres, stars, and rods. However, when we approach radioactive nanoparticles, there's a significant gap in the literature. Incorporating radioactive properties into nanoparticles introduces additional technical and logistical challenges, making such studies rarer and, consequently, more valuable. Handling radioactive nanoparticles demands specialized expertise and infrastructure, confining their research to specific settings. Thus, radioactive gold nanoparticles stand as a distinctive and significant contribution to nanotechnological research 5,6. In many of these works, the common practice is to use commercial chloroauric acid as a precursor. However, this approach is not suitable for producing radioactive nanoparticles through neutron activation in a nuclear reactor, leading us to develop a unique synthesis route for this purpose 7. When venturing into nanoparticle synthesis, the choice of method varies according to the intended application.

Obtaining radioactive nanoparticles represents a particularly complex challenge when it comes to concentration. The relationship between activity-dose and concentration is a critical aspect in the synthetic process. While many studies reported in the literature demonstrate working with lower concentrations 8-9, our focus on higher concentrations demands a meticulous approach. In this scenario, the role of a stabilizing agent becomes crucial. It not only ensures the stability and integrity of the nanoparticles but is also vital for optimizing their biodistribution, maximizing their therapeutic efficacy while simultaneously mitigating potential adverse effects.

When conjugated with radioactive isotopes, AuNPs become potent therapeutic tools, capable of administering radiation directly to the tumor, shielding adjacent healthy tissues 10. The coating of AuNPs is crucial for optimizing their biodistribution and minimizing side effects. In this landscape, bovine serum albumin (BSA) stands out, not only for enhancing biocompatibility but also for its role in active tumor targeting, leveraging its affinity for specific cell receptors 11. This strategy becomes even more pertinent when considering the prevalence of prostate cancer, one of the leading malignancies diagnosed in men worldwide, underscoring the need for more effective therapies 12. In light of traditional treatments, such as surgery and chemotherapy, which often entail undesirable side effects, radioactively loaded BSA-coated AuNPs emerge as a promising innovation. This combination, melding albumin with AuNPs, amplifies the system's stability and selectivity, minimizes interaction with plasma proteins, and facilitates efficient targeting to tumor cells. Considering the photothermal attributes and NIR absorption of AuNPs, they position themselves as ideal candidates for therapeutic and theranostic interventions. Incorporating albumin as a coating safeguard these nano systems from degradation and undesirable interactions with bodily proteins 13.

The biodistribution analysis of nanoparticles *in vivo* is intricate but essential for deciphering their interaction with biological systems. Multiple factors can mediate the uptake of radioactive AuNPs in different tissues. This study will encompass the synthesis of BSA-coated AuNPs, *in vitro* assays evaluating the cytotoxicity of these nanoparticles in both radioactive and non-radioactive states, and therapeutic assessment in animal models with varying injected radioactive activities. The biodistribution investigation will map the location of the nanoparticles in various organs, validating their efficacy and safety. Additional aspects to be explored include the excretion routes of the nanoparticles and post-treatment hematological evaluation, shedding light on any systemic effects.

## Methods

### Gold Nanoparticles (^198^AuNPs)

Gold, used for neutron activation in the EA-R1 nuclear reactor at the Institute for Energy and Nuclear Research (IPEN), underwent analyses in the Hyper Pure Germanium detector to determine its isotopic purity. The gold sample was provided by the OUROMINAS company. The reagents used in the study include nitric acid (HNO3 PA - Synth), hydrochloric acid (HCl 37% PA - Synth), pearl sodium hydroxide (NaOH PA - Exodo), sodium citrate PA (CRQ), gum arabic (Synth), polyethylene glycol 2k (Laysan Bio), and bovine serum albumin (BSA - Sigma Aldrich 9048-46-8, molecular mass 66kDa). The water used throughout the process is of nanopure nature, surpassing MilliQ standards, as evidenced by the absence of nanometric bodies in DLS (Dynamic Light Scattering) analyses. All glassware and utensils used were previously cleaned with aqua regia and subsequently with nanopure water.

### Functionalization of Gold Nanoparticles with BSA

In situ preparation of chloroauric acid from previously irradiated and activated gold is termed as the synthesis of the mother solution, as reported previously 7. This synthesis is performed in a sealed chemical reactor which was previously purged with a water filter to prevent the entry of impurities. The design of this reactor aims to contain radioactive gases, channeling them directly into the exhaust hood filters. Inside a 50 mL round-bottom flask heated to 100°C, 97 µL of chloroauric acid solution (3 x10^-2 M) and 97 µL of gum arabic (5.4 x 10^-5 M) are introduced, under vigorous stirring. Subsequently, 10 µL of NaOH (14M) is added and, after a 30-second interval, 230 µL of sodium citrate (1M) is added to the system, leading to the formation of nanoparticles within seconds. After continuous stirring for 3 minutes, 33 µL of PEG (0.1 M) is added. Two minutes later, heating is discontinued, and the mixture is cooled. Upon reaching room temperature (25°C), 33 µL of BSA is introduced with intense stirring. After an additional 10-minute stirring, the nanoparticles are ready for use.

### Characterization

The characterization of the radioactive material was conducted following a meticulous standardization of the method. The route using non-radioactive material was exhaustively characterized using DLS (Dynamic Light Scattering) and TEM (Transmission Electron Microscopy - JEM-2100 Jeol model with coupled EDS). For the radioactive material, characterization was limited to the use of DLS, with results being compared to the non-radioactive route. This provided a solid foundation to assert the characteristics of the radioactive material. It's worth noting that techniques like TEM are not viable for radioactive material due to contamination concerns. The concentration of the nanoparticles was deduced through statistical analysis of the microscopy data and the amount of gold used in the synthesis, resulting in an estimate of 5.4292*10^16 nanoparticles/mL.

### Assessment of ^198^AuNPs BSA Activity

To assess the activity of ^198^AuNPs BSA, we employed a *Capintec*, INC. CII CRC - 25R well counter, equipped with a semi-dry ionization chamber. This specific equipment, which was pre-calibrated for measuring the activity of ^198^Gold, was utilized for both *in vitro* and *in vivo* assays.

In the *in vitro* assays, we measured the total activity of ^198^AuNP BSA, obtaining values expressed in units per liter (µL). This allowed us to ascertain the activity level with which we were working during the experiments.

For the *in vivo* assays, we adopted a meticulous approach to measure the activity of ^198^AuNPs BSA. We measured the total volume in the syringe both before and after injection into the animals. This procedure enabled us to precisely calculate the injected activity in each animal, ensuring the accuracy and relevance of our experimental results.

### *In Vitro* Assays - Cytotoxicity Test of BSA-coated AuNPs

All cell lines used were acquired from the Rio de Janeiro Cell Bank (BCRJ). Prostate tumoral (LNCaP / PC3) non tumoral (RWPE-1), and umbilical endothelial (HUVEC) cell were kept in cell culture flasks (75cm2) with Opti-MEM® culture medium (Gibco - 31985070), supplemented with 5% Fetal Bovine Serum - FBS (Gibco - 10270106) and 1% solution containing antibiotic mix (penicillin / streptomycin, 10.000U / 10.000 µg / L) and antimycotic (Amphotericin B, 2.5µg / mL, Sigma-Aldrich - A5955). Flasks were placed in an incubator (37 ºC, CO2 5%) After reaching confluence, cells were detached using trypsin / EDTA solution (0,25% / 0,05M).

For cytotoxicity experiments, cells were seeded in 96-well plates in 5000 cells / 100 µL / well density and let to adhere for 24h in incubator. Then, culture media was replaced by fresh medium with diluted suspensions of AuNPs, radioactive or not, and toxicity controls (NaCl 0,045%: negative control; DMSO 10%: positive control). AuNPs suspensions were diluted on culture media in the following concentrations: 2.5, 5, 7.5, 10, 12.5, 15, 17.5, and 20 µL per 100 µL / well. Cells were incubated with test suspensions and controls by 6, 24 or 48 hours. All evaluations regarding concentrations were conducted in octuplicate.

At the end of the treatment incubation period, culture media of wells were replaced by 100 µL of dPBS - Dulbecco's phosphate-buffered saline (Gibco) and subsequently removed to wash free particles from cells. After this process, each well received 20 µL of the MTS/PMS solution (previously prepared as per the manufacturer's instructions for CellTiter 96® AQueous Non-Radioactive Cell Proliferation Assay (MTS)) diluted in 100 µL of culture medium and incubated 2 to 4 hours. Absorbance was measured (490nm) in a plate reader (Multiskan EX, Thermo Scientific).

### *In Vivo* Assays - Treatment with BSA-coated ^198^AuNPs

Animal Selection: The research utilized BALB/C Nude strain rodents, obtained from the IPEN (Institute for Energy and Nuclear Research) animal facility, following guidelines approved by the CEUA (Ethics Committee on the Use of Animals) under the number 243/19. Male mice aged between 8 and 12 weeks were selected, with an average weight of 25 grams. This selection aims to ensure uniformity in biological response and minimize variables unrelated to the therapeutic intervention.

The choice of the PC-3 cell line over the LNCaP cell line offers significant advantages for prostate cancer treatment studies with AuNPs BSA. PC-3 is known to be a more aggressive cell line, displaying faster tumor growth, which can expedite data collection in *in vivo* studies. Additionally, resistance to hormonal therapy, commonly associated with the LNCaP cell line, can be avoided, making PC-3 a preferred choice for research focusing on non-hormonal therapies. The heterogeneity of PC-3 can also be beneficial in reflecting the diversity of prostate tumors in human patients. Overall, choosing PC-3 is suitable when seeking a more aggressive and diversified model to investigate targeted therapies for prostate cancer.

Tumor Cell Line Selection and Inoculation: The PC-3 tumor cell line was used. Animals were inoculated with 5 x 10^6 cells subcutaneously in the upper region of the right hind paw, using a volume of 100 µL comprised of a solution made up of 50% extracellular matrix (Gibco™ Geltrex™ LDEV-Free, hESC-Qualified, Reduced Growth Factor Basement Membrane Matrix).

Experimental Groups: Once the tumors reached an average volume of 100 mm^3, the animals were divided into three experimental groups:

Group 1 (n=8): Received radioactive gold nanoparticles (^198^AuNPs BSA) with an average activity of 400 µCi.

Group 2 (n=8): Were administered an average activity of 500 µCi of the radioactive ^198^AuNPs BSA.

Control Group (n=6): Did not receive any radioactive treatment.

Biodistribution Study: Among the rodents in Groups 1 and 2, two individuals from each set were selected for the evaluation of the biodistribution of the ^198^AuNPs BSA at two temporal moments, respectively 3 and 24 hours post-administration. The choice of the 3 and 24-hour time points for biodistribution studies of ^198^AuNPs BSA is justified by the need to assess the initial distribution of nanoparticles in tissues, their presence in the bloodstream, and potential early accumulation (3 hours), as well as to examine long-term retention in target organs and determine whether nanoparticles continue to accumulate or are eliminated (24 hours). Furthermore, these time points allow for the evaluation of therapeutic effects at different stages, identification of organ-specific distribution patterns, and compliance with relevant regulations.

Nanoparticle Administration: The mouse was anesthetized with an inhalation anesthetic (Isoflurine - Cristália). The administration was carried out intratumorally, with each animal receiving an aliquot of **30 µL.**

Tumor Monitoring: After the administration of the AuNPs, tumor volumes were evaluated bi-weekly for a period of 21 days using a digital caliper.

Housing Conditions: Animals were housed in specific cages, under controlled environmental conditions. They had free access to water and feed, following recommended standards.

Before initiating our trial, the animals selected for treatment were microchipped. The use of microchips in animals prior to effectiveness trials is essential to ensure traceability and individualized monitoring of the experimental subjects. These devices enable accurate data collection, individual identification, and treatment history, optimizing result validation and ensuring the integrity of studies involving gold nanoparticles (AuNPs) or other interventions.

### Biodistribution Studies of BSA-coated ^198^AuNPs in Animal Models

Following the previously described protocol regarding the therapeutic efficacy evaluation of BSA-coated AuNPs, the animals were prepared in an identical manner. Four animals were selected, with 2 representing each treatment group. Each animal was properly identified, microchipped, and subjected to the therapeutic protocol as previously outlined.

After the administration of the nanoparticles, two distinct temporal intervals were established for biodistribution analysis: 3 hours post-administration and 24 hours post-administration. Within each group, one animal was designated for the 3-hour temporal window and another for the 24-hour interval.

At the stipulated analysis interval, the animals were anesthetized with isoflurane, and a 30 µL blood sample was collected via the retro-orbital method. The harvested organs (Blood, Heart, Lung, Liver, Kidneys, Gallbladder, Spleen, Stomach, Small intestine, large intestine, Pancreas, Bone, Muscle, Brain, Fat, Bladder and Tumor) stored in labeled tubes and subsequently quantitatively analyzed with a Gamma counter (Gama-2470 Automatic Gamma Counter by Perkin Elmer) to assess the biodistribution of the nanoparticles (%ID/g). This protocol was repeated for all animals at the determined times.

### Hematological Profile Evaluation After BSA-Coated AuNPs Treatment

At the end of the treatment period with BSA-coated AuNPs, the hematological profile of 7 mice was evaluated to assess their blood conditions. Following the completion of all analyses related to therapeutic efficacy and prior to euthanasia, the animals were anesthetized with isoflurane. Approximately 300 µL of blood was collected from the animals for analysis. This sample was then placed in appropriate, pre-labeled tubes provided by the laboratory, containing EDTA as an anticoagulant.

The samples were promptly sent to LAB&VET Diagnostic and Veterinary Consultancy for hematological analysis. The leukocyte differential count technique was utilized, making use of the ABC PENTRA 80 device from HORIBA. The report was signed and validated by Dr. Naiadi A. Publio, CRMV-SP 32904.

### Statistical Analyses

All statistical calculations were conducted using Microsoft Excel Version 16.76 and GraphPad Prism 9.5.0 (GraphPad Software, Inc., La Jolla, CA, USA). Data were analyzed using one-way analysis of variance (ANOVA) followed by the Bonferroni test. A p-value less than 0.05 was considered statistically significant (*p < 0.05, **p < 0.01, ***p < 0.001, and ****p < 0.0001).

## Results

### Synthesis of BSA-Coated Gold Nanoparticles (AuNPs BSA)

**Figure 1:**
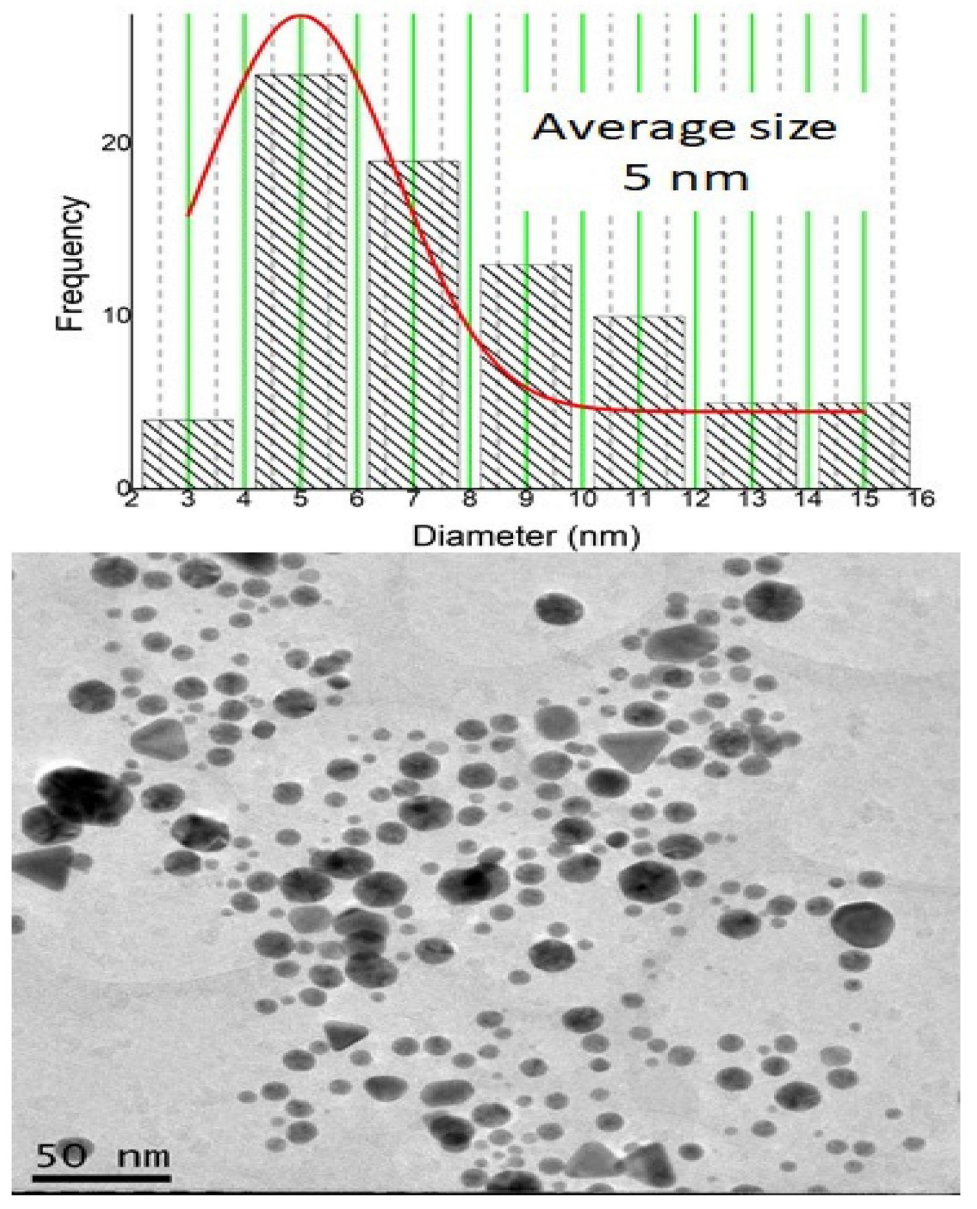
Image obtained by transmission electron microscopy (TEM) using a JEM-2100 JEOL microscope with attached energy-dispersive X-ray spectroscopy (EDS) of gold nanoparticles (NPsAu). The nanoparticles have an average diameter of 5 nm.

The produced BSA-coated gold nanoparticles have an average diameter of 5 nm, as illustrated by Figure 1 provided by the TEM equipment. Given this diameter, it's possible to calculate the volume of a single spherical nanoparticle using the formula V=4/3 πr^3.

Assuming a radius of 2.66 nm (half of the diameter), the calculated volume is approximately 65.45 nm^3.

The radius of an individual gold atom is 0.166 nm, giving it a volume of about 0.70 nm^3. Hence, we can estimate that each nanoparticle is composed of approximately 93 gold atoms by dividing the nanoparticle volume by the gold atom volume.

Additionally, the concentration of the mother solution was 0.03 M. The concentration of sample 1 was 3.15 x 10^19 gold nanoparticles per microliter, with a total activity of 400 µCi. The second produced sample had a concentration of 4.73 x 10^19 gold nanoparticles per microliter, with a total activity of 600 µCi.

### In Vitro Assays

#### Evaluation of the Cytotoxicity of Non-radioactive BSA-Coated Gold Nanoparticles (AuNPs BSA)

**Figure 2:**
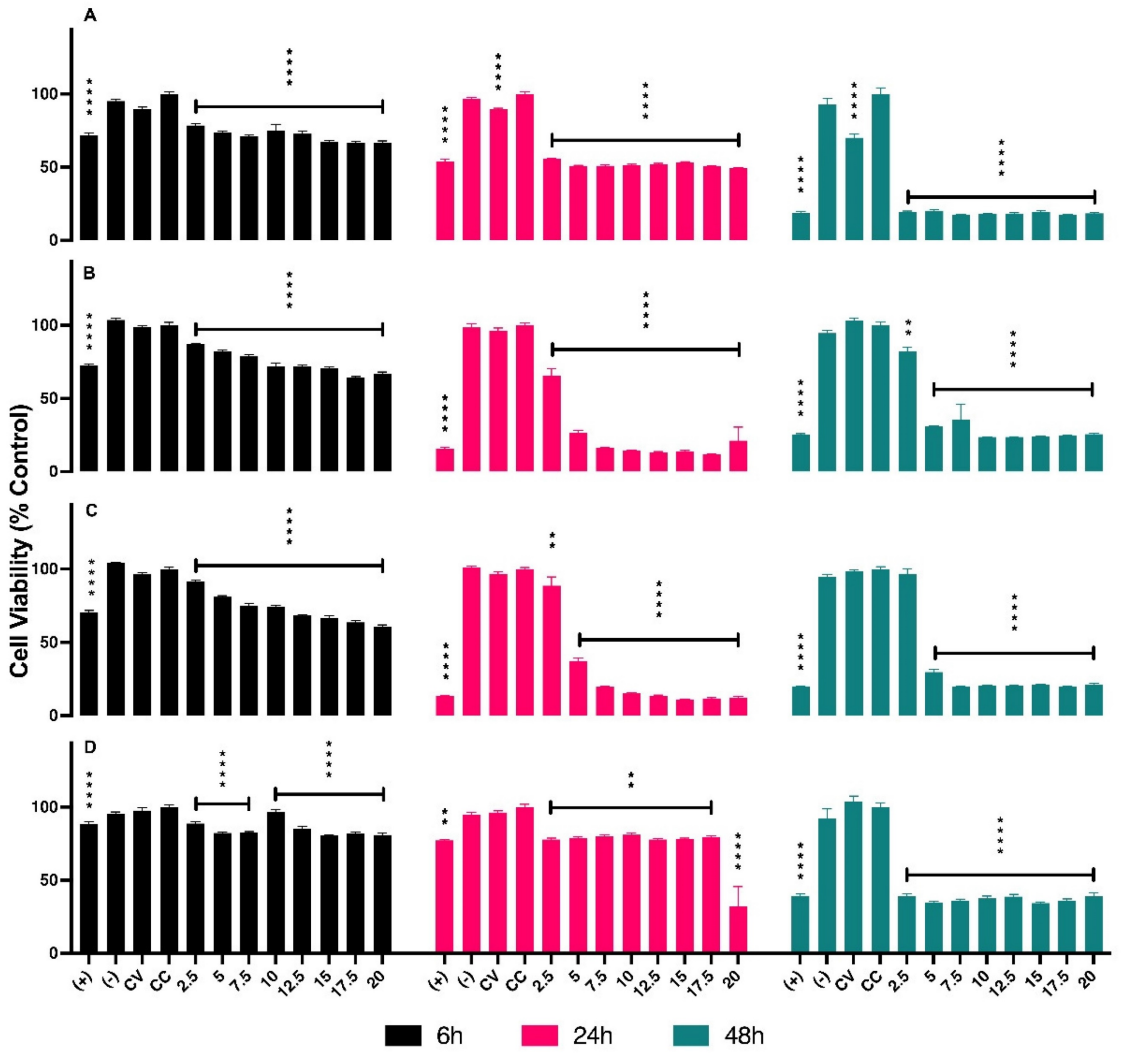
** Results of cytotoxicity assays of non-radioactive AuNPs BSA in PC-3, LNCaP, RWPE-1, and HUVEC cell lines.** Asterisks denote statistically significant differences compared to cell control (CC): * P < 0.005, ** P < 0.005, *** P < 0.004, **** P < 0.001. In the graphs Black, pink, and green bars represent evaluations at 6 hours, 24 hours, and 48 hours, respectively. **Graph A (PC-3):** In 6 hours (bars black) show cytotoxicity of 22% at 2.5 µL and 34% at 20 µL. Pink bars (in 24 hours) indicate 45% at 2.5 µL and 51% at 20 µL. Green bars reveal 80% cytotoxicity at all concentrations after 48 hours. **Graph B (LNCaP):** In 6 hours display cytotoxicity of 13% at 2.5 µL and 34% at 20 µL. In 24 hours demonstrate an increase to 35% at 2.5 µL, reaching 89% at 17.5 µL. Green bars (48hours) show 18% cell damage at the lowest concentration, reaching 70% from 5 µL onwards. **Graph C (RWPE-1):** Black bars (6 hours) indicate cytotoxicity of 9% at 2.5 µL, rising to 40% at 20 µL. Pink bars (24 hours) show over 60% of cells affected at the lowest concentration, increasing with dosage. In 48 hours present an average of 80% cytotoxicity for all concentrations. **Graph D (HUVEC):** Black bars (6 hours) reveal cytotoxicity of 12% at 2.5 µL, slightly increasing with dosage. Pink bars (24 hours) show fluctuation in cytotoxicity between 19% and 23%. Green bars indicate a variation of 61% cytotoxicity between the lowest and highest concentration.

**Figure 3:**
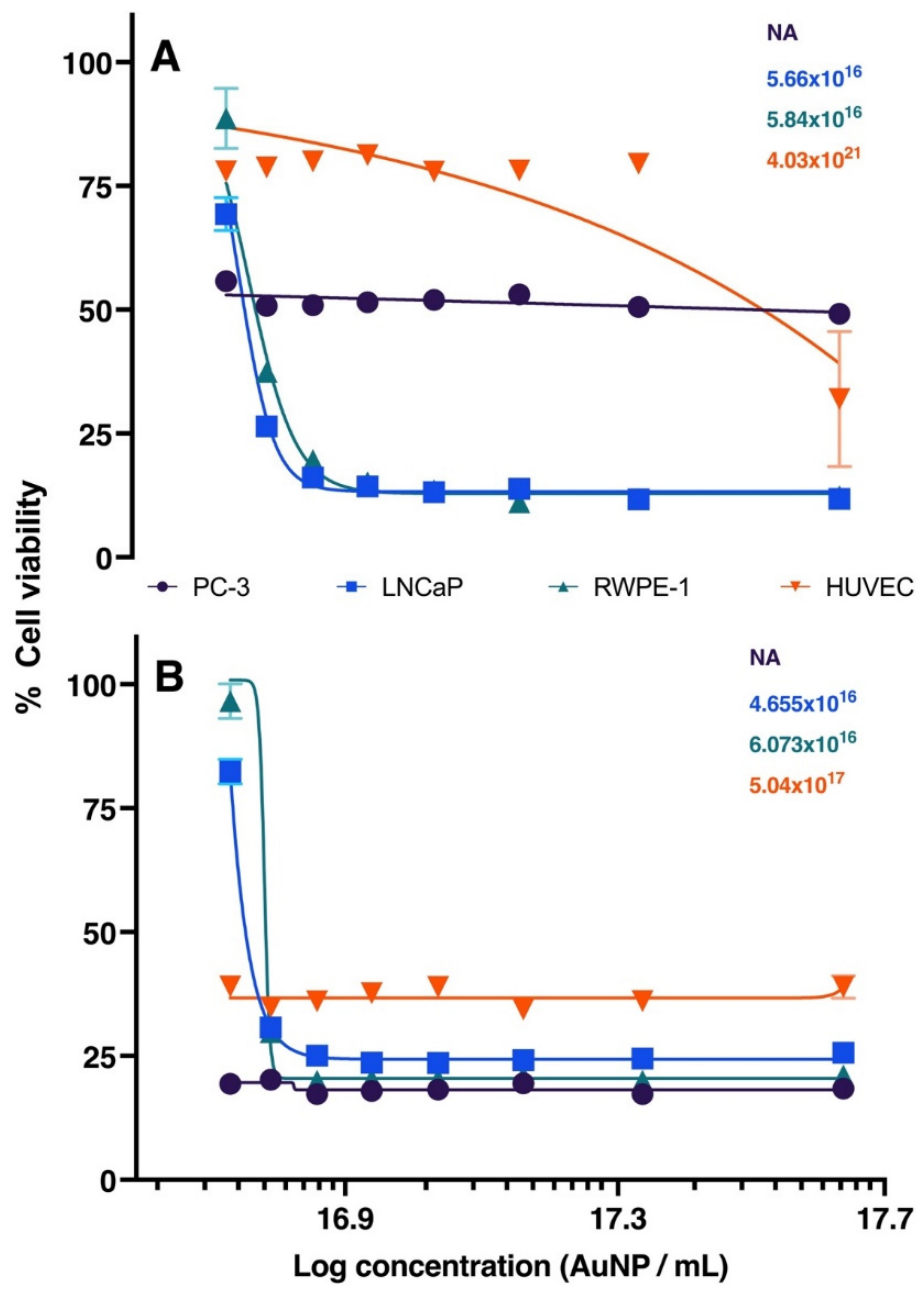
** Graphic representation of IC50 after 24 and 48 hours of treatment in PC-3, LNCaP, RWPE-1, and HUVEC cell lines.** Black, blue, green, and red lines represent PC-3, LNCaP, RWPE-1, and HUVEC, respectively. **Graph A (24 hours):** Highlights the primary effect at the concentration of 20 µL. LNCaP (blue line) reaches IC50 with 2.9 μL of AuNPs BSA. RWPE-1 (green line) shows IC50 at 3.6 μL of AuNPs BSA. HUVEC (red line) requires 21 μL to reduce cell viability by 50%. **Graph B (48 hours):** Displays results after extended treatment. PC-3 (black line) shows a similar trend. LNCaP (blue line) has an IC50 at 3.6 μL. RWPE-1 (red line) indicates 4.6 μL to affect 50% of cells. HUVEC (green line) demonstrates that 1.7 µL of nanoparticles reduce cell viability by half.

The concentrations of AuNPs BSA, in µL, used for *in vitro* assays are presented in Table 1. The series of graphs shown in Figure 2 depict the cytotoxicity of cells exposed to non-radioactive AuNPs BSA. The evaluated cell lines include PC3 (Graph A), LNCaP (Graph B), RWPE-1 (Graph C), and HUVEC (Graph D), with the latter being used as a control group. The designated evaluation intervals were 6, 24, and 48 hours, and the concentrations of AuNPs BSA tested ranged from 2.5 to 20 µL (The corresponding values in concentrations of ^198^AuNPs BSA are expressed in Table 1). The IC 50 for each cell line is outlined in Figure 3, the IC50 values for the 24-hour evaluation period are presented no Graph A as follows: PC-3 - 1.847, LNCaP - 2.916, RWPE-1 - 3.659, and HUVEC - 20.92. In Graph B, the IC50 values for the 48-hour evaluation period are displayed as follows: LNCaP - 0.2253, RWPE-1 - 0.1758, and HUVEC - 0.4561.

#### Evaluation of the Cytotoxicity of Radioactive BSA-Coated Gold Nanoparticles (^198^AuNPs BSA)

As with the non-radioactive trials, studies were also conducted using radioactive nanoparticles on the same cell lines. During the plating phase, we adopted a distinct approach to mitigate possible interferences from irradiation between the wells. A separate plate was exclusively used for controls, which included: Positive Control (DMSO), Negative Control (NaCl), and Cell Control (CC). This strategy aimed to ensure that radiation emitted from the treated wells did not compromise the development of controls. The vehicle control test was not conducted in this context since it had already shown satisfactory efficiency in the non-radioactive trials. In the treatment microplate, three distinct concentrations of AuNPs BSA were tested: 2.5, 5, and 7.5 µL. Additionally, the treated wells were spaciously distributed to minimize any influence from adjacent radiation.

After conducting the non-radioactive cytotoxicity study, it was observed that concentrations above 7.5 µL of AuNPs proved cytotoxic to the cells. Therefore, for the radioactive assay, concentrations of 2.5, 5, and 7.5 µL of AuNPs were chosen, ensuring that any observed cell damage could be attributed to radiation and not to the cytotoxicity of the nanoparticles. The same controls used in the non-radioactive assay were employed in the radioactive assay, except for the vehicle control, given its previously verified efficiency.

Cytotoxicity tests of radioactive BSA-coated gold nanoparticles (AuNPs BSA) were conducted on four types of cells: PC-3, LNCaP, RWPE-1, and HUVEC. The tested concentrations were 0.8, 1.6, and 2.4 µCi. The results showed that: For PC-3, after 6 hours of treatment, the percentages of cell death were 18% (0.8 µCi), 9% (1.6 µCi), and 17% (2.4 µCi). After 24 hours, the percentages were 30% (0.8 µCi) and 20% (2.4 µCi). After 48 hours, the percentages were 5% (0.8 µCi) and 1% (2.4 µCi). For LNCaP, after 6 hours, the percentages of cell death were 15% (0.8 µCi) and 9% (2.4 µCi). After 24 hours, the percentages were 26% (0.8 µCi) and 28% (2.4 µCi). After 48 hours, the percentages were 2% (0.8 µCi) and 30% (2.4 µCi). For RWPE-1, no cell death was observed at any time point (0.8, 1.6, and 2.4 µCi).

**Figure 4:**
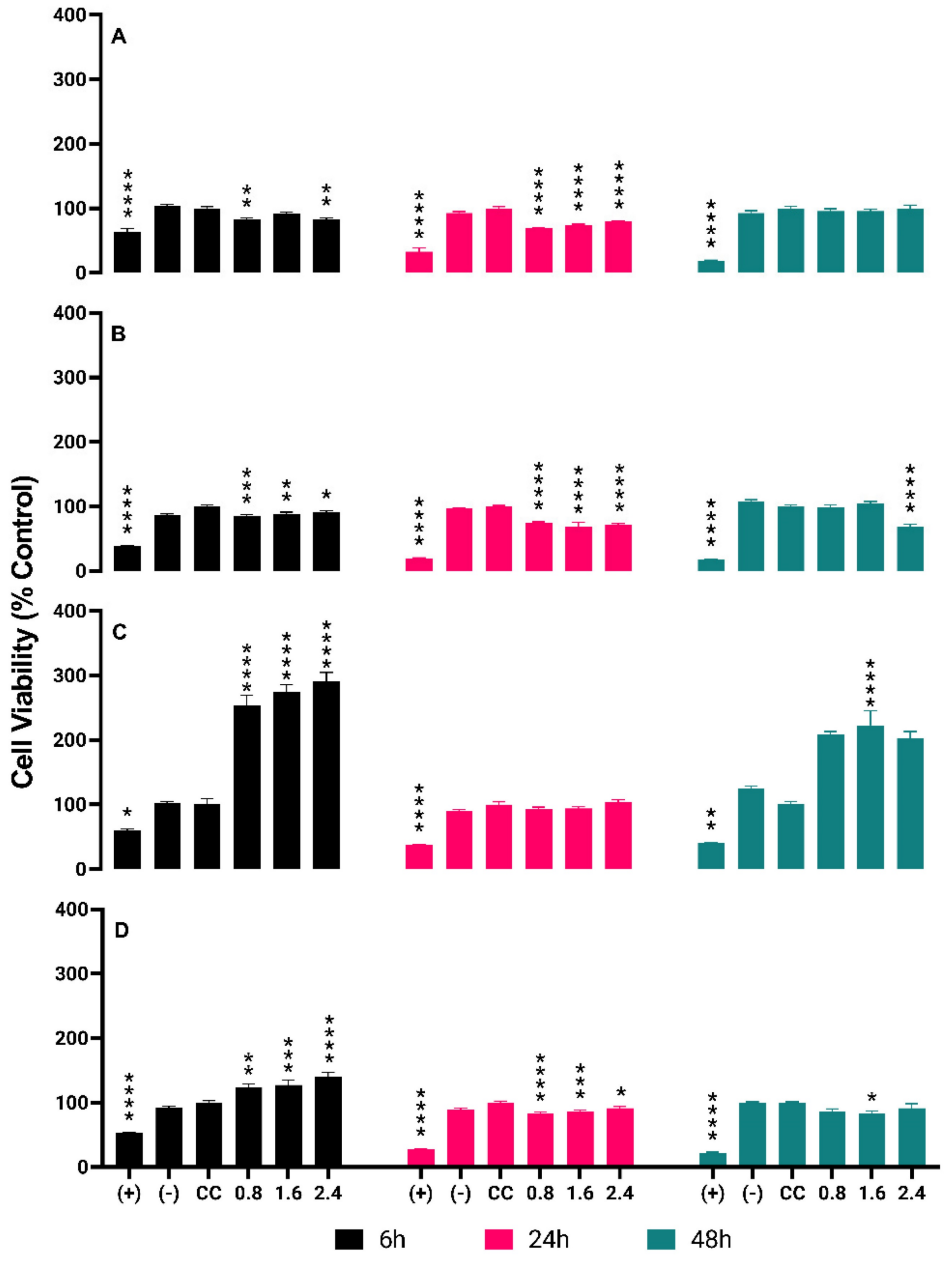
** Detailed Cytotoxicity Response of ^198^AuNPs BSA in the Cell Lines PC-3, LNCaP, RWPE-1 e HUVEC Over Time.** Asterisks denote statistically significant differences from the cell control (CC): * P < 0.005, ** P < 0.005, *** P < 0.004, **** P < 0.001. Black bars represent 6-hour results, pink bars for 24 hours, and green bars for 48 hours. **Graph A (PC-3 cells):** Cytotoxicity at 0.8 µCi starts at 18% and drops to 5% over 48 hours. Similar trends are observed at 1.6 µCi (9% to 1%) and 2.4 µCi (17% to 1%), indicating a time-dependent decrease in cytotoxicity across all concentrations. **Graph B (LNCaP cells):** Initial cytotoxicity of 15% at 0.8 µCi decreases to 9% at the highest concentration after 6 hours. After 24 hours, cytotoxicity slightly increases, reaching 28% at the highest dose, but then drops markedly to 2% at 48 hours. **Graph C (RWPE-1 cells):** Shows an opposite trend with cell proliferation observed at all tested concentrations. Proliferation rates increase over time, reaching up to 103% at 24 hours and a striking 222% after 48 hours at the highest concentration. **Graph D (HUVEC cells):** Demonstrates an initial increase in cell proliferation at 6 hours (up to 40% at the highest dose) followed by a decrease in cytotoxicity after 24 and 48 hours, particularly at higher concentrations, with the highest dose showing only 9% cytotoxicity after 48 hours.

For HUVEC, after 6 hours, no cell death was observed. After 24 hours, the percentages of cell death were 17% (0.8 µCi), 14% (1.6 µCi), and 9% (2.4 µCi). After 48 hours, the percentages of cell death were 14% (0.8 µCi) and 9% (2.4 µCi). These results are detailed in Figure 4 in Graphs A, B, C and D.

### *In Vivo* Assays

#### Biodistribution of BSA-Coated Gold Nanoparticles (^198^AuNPs BSA) 3- and 24-Hours Post-Injection

**Figure 5:**
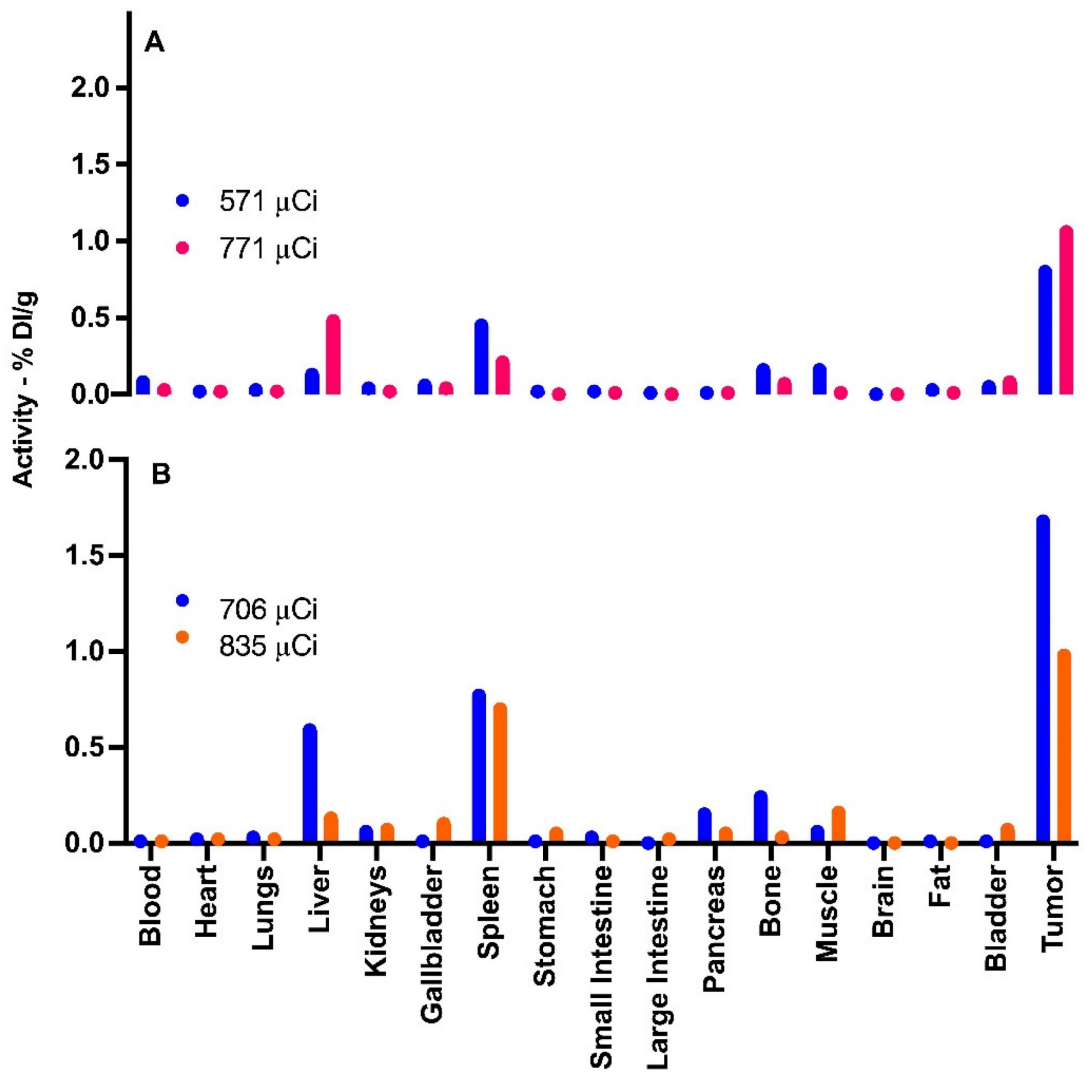
** Biodistribution of ^198^AuNPs BSA in Mice Post-Treatment. Graph A (3 hours post-treatment):** Blue bars represent the biodistribution in an animal treated with a total activity of 571 µCi, showing detection primarily in the liver, spleen, bones, muscle, bladder, and notable uptake in the tumor. Orange bars illustrate the results in an animal subjected to 771 µCi, demonstrating predominant uptake in the liver, spleen, bones, bladder, and tumor, in line with predictions. **Graph B (24 hours post-treatment):** Blue bars correspond to an animal that received 706 µCi of total activity, exhibiting uptake in the liver, spleen, pancreas, bones, muscle, with significant capture in the tumor. Orange bars indicate an animal administered with 835 µCi, showing standard uptake in the liver, gallbladder, spleen, discreet uptake in the pancreas, bones, muscle, and bladder, and similarly to the blue group, elevated uptake in the tumor.

Figure 5 provide data from the biodistribution assays conducted on four distinct experimental group mice. The Graph A showcases the biodistribution results in two chosen animals in 3 hours pos treatment. The mouse represented by the blue line was administered an activity of 571 µCi, whereas the mouse denoted by the orange line received an activity of 771 µCi.

In contrast, Graph B depicts the findings from mice that were given doses of 706 µCi (represented by the blue line) and 835 µCi (illustrated by the orange line), with assessments conducted 24 hours after injection. The choice to employ these higher µCi activities in mice for the biodistribution study arose since these particular animals received slightly above-average activities. Hence, we chose to utilize them for this specific study, while reserving other animals for therapeutic efficacy evaluations.

By looking into the biodistribution at these distinct intervals post-administration, we gain a clearer understanding of the dispersal and concentration of the nanoparticles within the biological system. This aids in painting a comprehensive picture of their potential therapeutic benefits and their safety profile.

#### Therapeutic Efficacy of BSA-Coated Gold Nanoparticles

In Table 2, the outcomes from the examination of 12 mice treated with ^198^AuNPs BSA over a span of 21 days are presented. The table delineates the radioactive activity administered in each subject, as well as initial tumor volumes and observed volume changes post therapeutic intervention. Throughout the stipulated period, animals were bi-weekly monitored, furnishing an accurate assessment of tumor progression.

**Figure 6:**
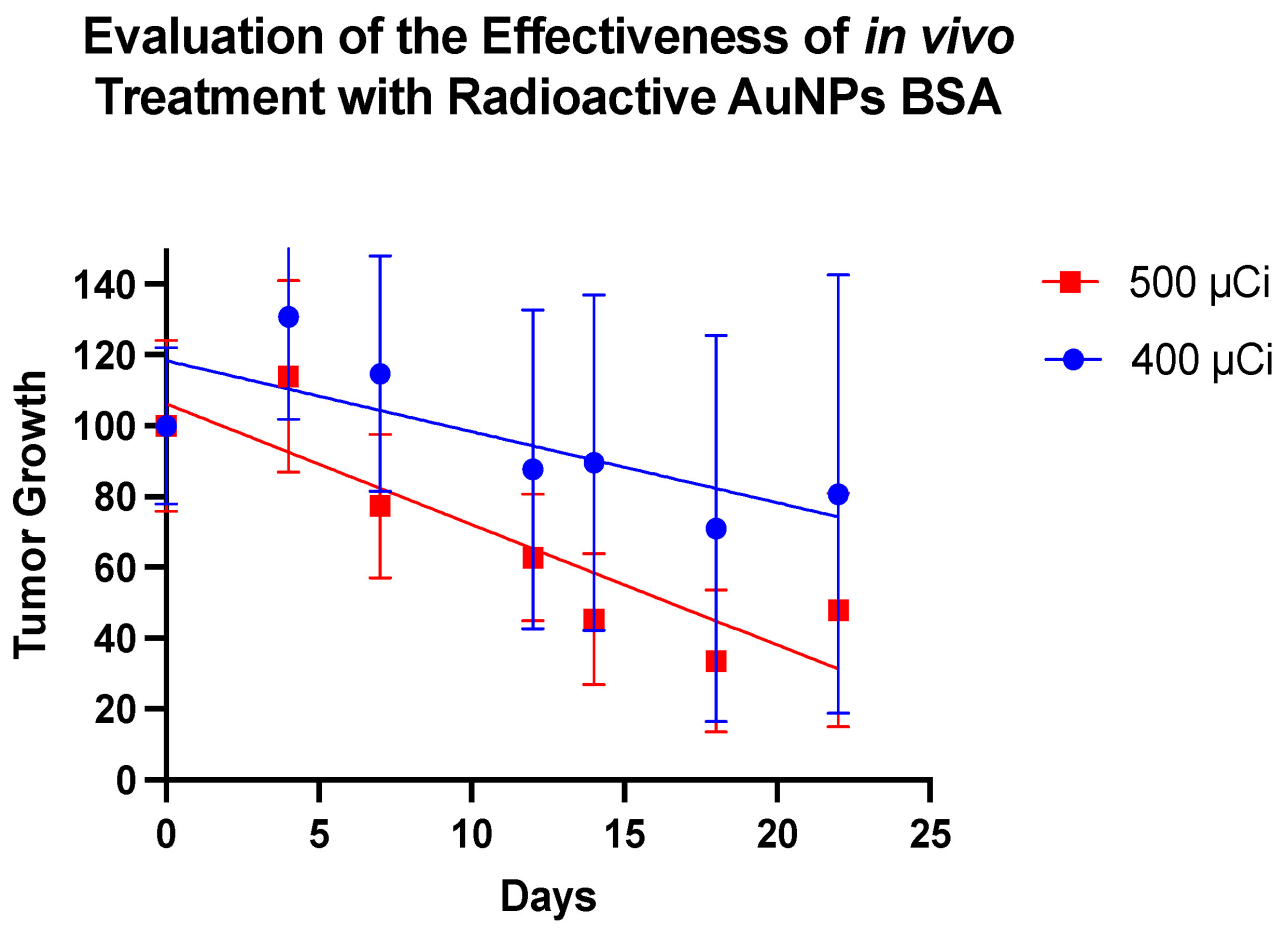
The graph outlines the therapeutic efficacy of BSA-coated gold nanoparticles in two distinct groups of animals, monitored over 22 days. The representation in blue belongs to Group 1, subjected to an average dose of 400 µCi, resulting in an average tumor decrease of 30%. In contrast, the representation in red corresponds to Group 2, treated with an average dose of 500 µCi, showing a tumor reduction of 70%.

**Figure 7:**
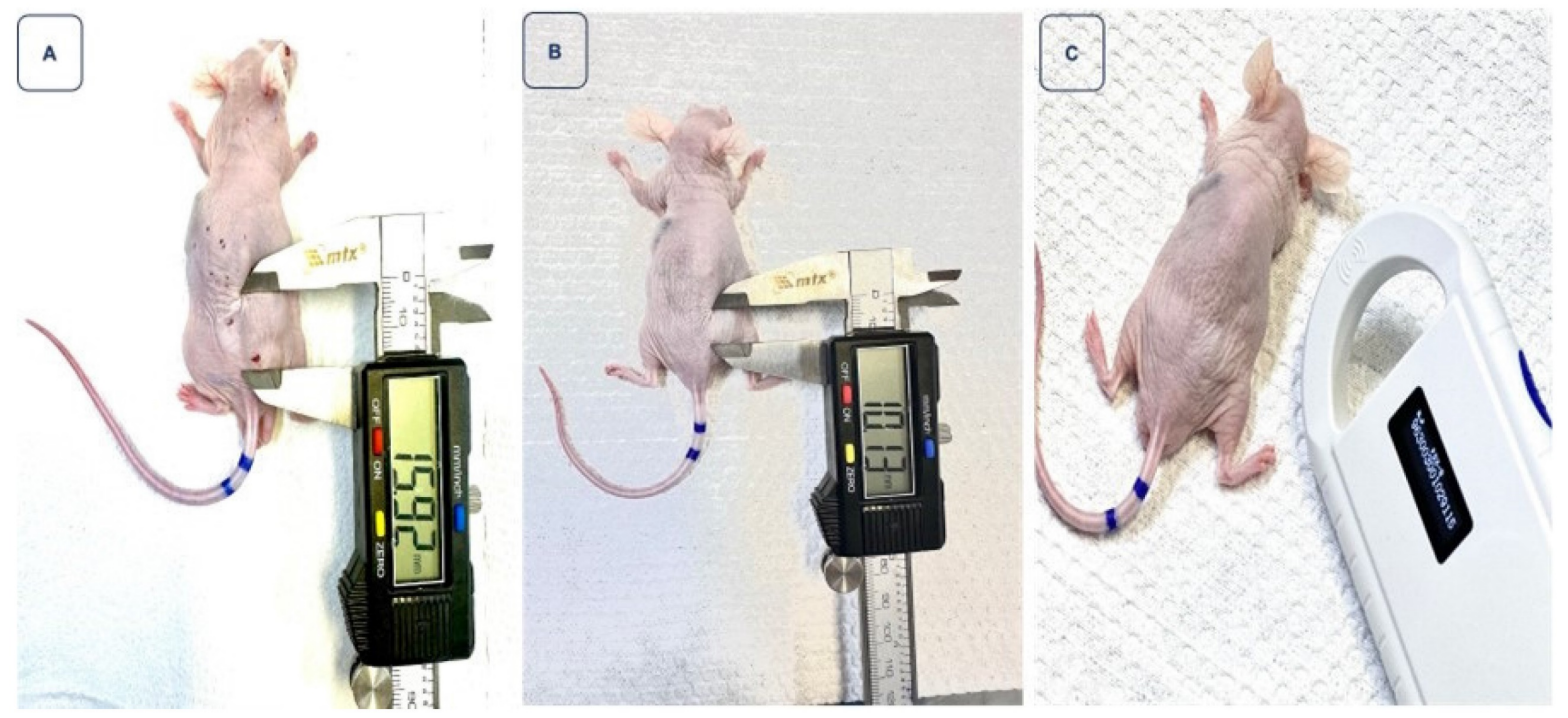
Monitoring of tumor regression in animal 2, ID 9115, after receiving 417 µCi of BSA-coated AuNPs. **Image A** illustrates the tumor measurement on the day of treatment, with a volume of 107.63 mm³. In **image B**, a reduction in volume is observed on the seventh day after treatment. Finally, **image C** highlights that on the 14th day, the reduction was so significant that measurement with a digital caliper became unfeasible.

Upon treatment with the nanoparticles, clear changes in the tumor masses were discernible. It is noteworthy that Group 2 appears to demonstrate a more effective therapeutic response (Figure 6). Figure 7 depicts the trajectory of tumor volume reduction from the outset, spanning the seventh day and extending to the fourteenth day post-treatment, at which point the mass approached zero. Furthermore, with an initial volume of 107.63 mm³, a significant reduction in volume was observed on the seventh day after treatment, to the extent that measurement with a digital caliper became unfeasible.

**Figure 8:**
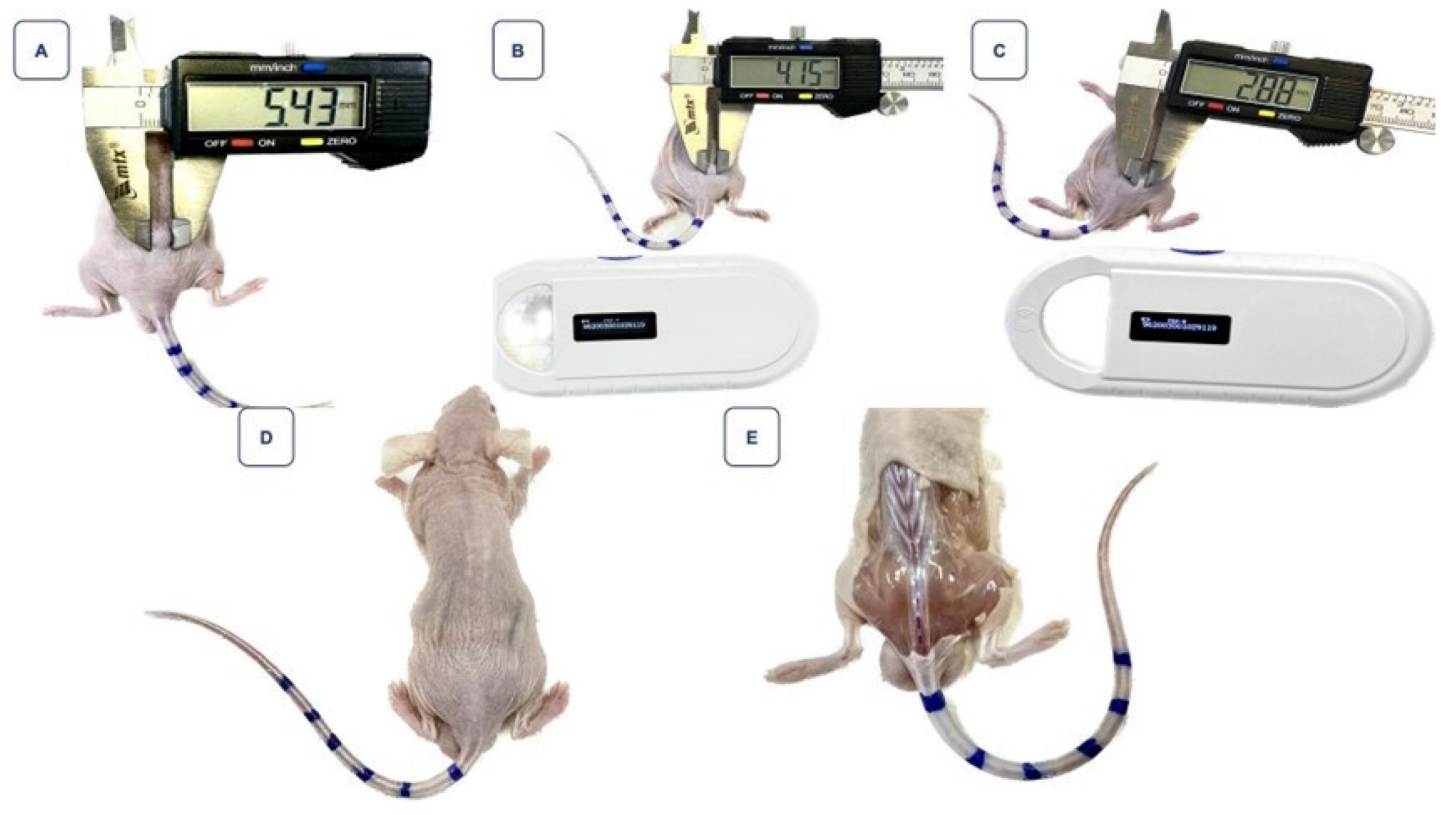
Monitoring of tumor regression in the mouse identified as animal 6, ID 9119, after treatment with 268 µCi of BSA-coated AuNPs. **Image A:** Represents the tumor on the day of intervention, with a volume of 16.58 mm³. **Image B:** Shows a reduction in tumor volume on the seventh day post-treatment. **Image C:** On the 14th day, there is a noticeable continued reduction in the tumor. **Image D:** By the 18th day, the regression of the tumor is so significant that measuring it with a digital caliper becomes impractical. **Image E:** No evidence of the tumor is visible under the mouse's skin.

Figure 8 highlights the volume reduction in another mice, starting with an initial volume of 16.58 mm³, which also exhibited complete regression, marked by the absence of visible tumoral tissue beneath the skin.

It's worth noting that, while these results are preliminary, the application of BSA-coated gold nanoparticles showcases a promising path for targeted therapeutic interventions in cancer treatments. Further research and optimizations could pave the way for a revolutionary approach in managing malignancies.

#### Hematological Profile Analysis Post ^198^AuNPs BSA Treatment

Following the ^198^AuNPs BSA treatment, the hematological data of the mice treated with radioactive gold nanoparticles coated with BSA was meticulously analyzed against the known literature. A detailed examination of the seven animals reveals notable conclusions.

##### Red Blood Cells and Red Series

Five out of the seven mice had erythrocyte counts that were within or slightly below the reference range. This data is encouraging since only slight variations were observed, suggesting that the therapy with ^198^AuNPs BSA might have a minimal impact on the production or maintenance of erythrocytes.

Regarding hemoglobin and hematocrit levels, most animals maintained levels near the reference interval, with only a few values slightly below normal.

The cellular morphology of the red series remained normal in the majority of the animals, with only two of them showing mild anisocytosis and polychromasia. This is noteworthy, especially considering that these animals underwent oncological treatment.

##### Leukocytes and White Series

Leukocyte levels varied, with some animals showing significantly elevated values compared to the reference range. While the cause of this elevation is not immediately clear, it's important to consider potential inflammatory effects or immune responses associated with the treatment. Notably, observations of neutrophilia, such as hyper-segmented neutrophils and atypical nuclear shapes, were identified in some animals. These changes may indicate adaptive responses to the treatment.

##### Platelets

Platelet counts remained largely within or slightly above the reference range, suggesting adequate maintenance of coagulation and related functions even after exposure to AuNPs BSA.

##### Hemoparasites

All animals tested negative for hemoparasites, a promising indication that exposure to ^198^AuNPs BSA did not increase susceptibility to parasitic infections in the blood.

To evaluate the hematological consequences of ^198^AuNPs BSA treatment, specific and relevant data were collected from a complete blood count, focusing on items selected for our analysis, allowing a detailed view of the hematological profile of the mice post-treatment, as illustrated in Table 3.

## Discussion

The production of radioactive gold nanoparticles - BSA represented a considerable innovation in the field of nanomedicine towards oncological therapies. By combining the unique properties of gold in terms of surface plasmon resonance with its radioactivity and the biochemical functionality of BSA, we obtained a nanomaterial with distinct characteristics.

The synthesis method adopted ensured the generation of consistent and reproducible nanoparticles. Although the radioactive nature of these nanoparticles presents challenges for their characterization - with the impracticality of traditional techniques such as TEM - the use of DLS and the comparison with the non-radioactive route ensured solid confidence in the quality of the material produced.

With this rigorous characterization, the nanoparticles proved suitable for use in both *in vitro* and *in vivo* studies.

Kadhim and his team investigated the toxic potential of GNPs using both in vitro and *in vivo* approaches. Their studies revealed that, when used in reduced concentrations, GNPs are biocompatible in both *in vitro* and *in vivo* settings, reinforcing the safety of AuNPs for human use. With the advancement of research involving AuNPs, human exposure to these nanoparticles has increased, making safety assessments foundational 4; 14.

The assessment of cytotoxicity is a foundational pillar in biological and pharmaceutical research, offering an initial indication of the efficacy and safety of potential compounds 12. In the present study, the cytotoxicity of non-radioactive AuNPs BSA was examined on the PC-3, LNCAP, RWPE-1, and HUVEC cell lines. A notable variability in the cytotoxic response of the cell lines concerning exposure to AuNPs BSA was observed.

Of particular note are the prostatic tumor cell lines PC-3 and LNCAP. These showed considerable sensitivity to the cytotoxicity of AuNPs BSA, especially at higher concentrations and longer exposure times. Such findings are promising and indicate a potential therapeutic use of AuNPs BSA in prostate cancer treatment.

In contrast, the RWPE-1 cell line, representing normal prostate cells, also showed sensitivity to the nanoparticles. While this observation is of some concern, it underscores the urgent need to optimize nanoparticle dosage and delivery specificity. This is done to maximize their efficacy against tumor cells and minimize adverse effects on healthy cells.

As for the HUVEC cell line, frequently used as a control in cytotoxicity assays, the results serve as a comparative parameter to understand the specificity and toxicity of nanoparticles in different cellular contexts.

The dose-dependent response evidenced in specific cell lines is a characteristic widely documented in toxicological studies, with the cellular response intensifying with the increase in agent concentration 15. This suggests that non-radioactive AuNPs BSA may have an intracellular mechanism of action that intensifies according to the applied dosage.

The use of gold nanoparticles (AuNPs) coated with bovine serum albumin (BSA) as potential therapeutic agents has been gaining prominence in the landscape of oncological nanomedicine 16. In this study, we evaluated the cytotoxicity of radioactive AuNPs BSA in four distinct cell lines, at activities of 0.8, 1.6, and 2.4 µCi over three different incubation periods.

In prostatic tumor cells, PC-3 and LNCAP, a cytotoxic effect was evident at various concentrations and evaluation times. The PC-3 and LNCAP cell lines demonstrated variable sensitivities to AuNPs BSA, being more sensitive in the initial hours of treatment, with a decrease in cytotoxic efficacy as the incubation time extended. These findings corroborate previous studies that showed that gold nanoparticles can interact differently with tumor cells, possibly due to differences in absorption, metabolism, and damage response mechanisms 17.

Surprisingly, in RWPE-1 cells, which are normal prostatic cells, and in endothelial HUVEC cells, we observed proliferation phenomena at certain concentrations and times. The induced cell proliferation can be attributed to several mechanisms. One possible mechanism might be the stimulation of signaling pathways that promote cell proliferation, such as the PI3K/AKT pathway, which has been previously demonstrated with nanoparticles in certain contexts 18. Additionally, the controlled release of radioactive agents might lead to an adaptive response that stimulates cell proliferation in a phenomenon known as radiation hyper-response 19.

The BALB/c Nude mouse lineage, characterized by its immunodeficiency, is widely recognized in oncological research. Their inability to reject grafts, including human tumor cells, makes it a robust model for tumor assessment. The biodistribution of nanoparticles, like the ^198^AuNPs BSA, is complex and can be modulated by multiple factors, among them the animal's own lineage, the injection site, and nanoparticle characteristics, such as size and coating.

Our research underscores the influence of the mononuclear phagocyte system (MPS), especially the liver and spleen, in the biodistribution of ^198^AuNPs BSA. The attraction of these nanoparticles to phagocytic cells, such as macrophages, points to the significance of these organs in the sequestration and processing of these particles. This phenomenon is supported by the evident accumulation of nanoparticles in the liver and spleen observed in our assays. Moreover, the presence of nanoparticles in the bladder suggests potential excretion routes, and the observation of uptake in muscles and bones underscores the need for a comprehensive biodistribution analysis 20, 21.

The uptake of gold nanoparticles in muscular and bone tissues, even if minimal, emphasizes the multifaceted interaction of these nanoparticles with various tissues and cells. The phagocytosis process and the presence of osteoblasts and vascularized bone marrow provide additional insights into potential interaction mechanisms 22.

The therapeutic efficacy of ^198^AuNPs BSA is evident. There is a clear trend indicating that higher dosages result in more robust therapeutic responses. However, it's worth recognizing that variability, whether related to the tumor itself or the host, might play a role in modulating these outcomes. Some animals exhibited responses that deviate from the expected pattern, illustrating the inherent complexity in this field of study.

Figure 7 stands as a clear demonstration of the significant regression of the tumoral mass, an observation of undeniable importance in the therapeutic context. In contrast, Figure 8 presents a particularly intriguing scenario. Despite starting with a smaller tumor size and receiving a relatively low dose of 268 µCi - a value below the average of other treated animals and unique in this study - the mouse exhibited complete tumoral regression. This response was so profound that, upon examining the injection site, no visible traces of tumoral tissue could be found beneath the animal's skin. This outcome, resulting from a lesser dose, emphasizes the need for further investigations regarding response variability and the potential optimization of the therapeutic dose.

Recognizing the achievements made, it is imperative to also emphasize the need to delve deeper into our investigations. Exploring the nuances and peculiarities of these variabilities can not only strengthen our understanding of the efficacy of ^198^AuNPs BSA but also pave the way for future optimizations in treatment.

The examination of the hematological profile post-treatment with gold nanoparticles coated with BSA (^198^AuNPs BSA) in mice inoculated with prostate tumor cells revealed noteworthy results. Hematology serves as a pivotal indicator of an organism's overall health, and alterations in this profile can provide invaluable insights into the biocompatibility and potential toxicity of a treatment.

Hematological assessment is a vital tool in the realm of translational research, especially when it involves the administration of emerging therapeutic agents, such as gold nanoparticles coated with BSA, in animal models. Animals inoculated with prostate tumor cells and subjected to experimental treatments might exhibit significant changes in hematological parameters.

Hematological monitoring is essential when evaluating the biocompatibility and efficacy of therapeutic agents, especially in innovative therapies based on nanotechnology, such as ^198^AuNPs BSA. These nanoparticles, given their ability to extensively interact with biological systems, demand a meticulous assessment of their potential impact on the hematological system 23.

A complete blood count provides insights into an organism's overall biological response to treatment. Elements such as erythrocyte count and hemoglobin levels are key indicators of the blood's capacity to carry oxygen. In most of the studied animals, erythrocyte levels remained within or close to the reference range, indicating that the oxygen-carrying capacity remained intact. This observation is bolstered by the normal erythrocyte morphology in many of the animals, suggesting that erythropoiesis was not adversely affected by the presence of ^198^AuNPs BSA 24.

The leukocyte count, another vital component of the blood count, is indicative of immune and inflammatory responses. In this study, variations in leukocyte levels were observed among the animals, with some displaying leukocytosis. Although leukocytosis can be a consequence of infections or inflammation, it can also be provoked by other factors, such as stress or medical treatments 24; 25. Given the experimental setup and the lack of evidence for other pathologies, it is postulated that the observed leukocytosis might be a transient reaction to ^198^AuNPs BSA treatment, and not necessarily an indicator of prolonged toxicity.

Additionally, platelets play a critical role in blood clotting. Significant changes in their count can indicate toxic impacts on bone marrow progenitor cells or clotting issues.

Encouragingly, the absence of hemoparasites in all animals suggests a good overall state of health during the experimental period, corroborating the potential safety of the nanoparticles in the context of this study.

## Conclusion

The synthesis of radioactive gold nanoparticles - BSA constitutes a significant milestone in oncological nanomedicine. Despite the characterization challenges posed by radioactivity, the developed methodology ensured the production of high-quality nanoparticles. Their meticulous preparation suggests that these nanoparticles are well-positioned and suitable for use in future experimental studies, paving the way for innovations in cancer treatment.

The cytotoxicity assay of the non-radioactive AuNPs BSA on the mentioned cell lines revealed cytotoxic potential, especially in the prostatic tumor cell lines PC-3 and LNCAP. The activity observed in RWPE-1 and HUVEC cells underscores the need for further investigations to optimize the compound's selectivity. Subsequent studies focused on the selective action of nanoparticles on tumor cells and minimizing their effects on normal cells and controls will be essential to confidently advance toward therapeutic applications.

The study of the cytotoxicity of the radioactive AuNPs BSA in the evaluated cell lines revealed promising results, especially in the context of tumor cell lines. However, the proliferation phenomenon observed in RWPE-1 and HUVEC cells suggests that a deeper investigation is needed to understand the underlying mechanisms and thus optimize the application of these nanoparticles. Considering the specificity required in cancer treatment, it's crucial that targeted therapeutic strategies are not only effective against tumor cells but also minimize adverse effects on healthy cells.

Future approaches might consider modifications in the design of the nanoparticles, aiming for better specificity, or the combination with other therapeutic agents, to maximize efficacy and minimize undesired effects.

Our research with BALB/c Nude mice served as a pivotal platform for investigating the biodistribution and therapeutic efficacy of BSA-coated gold nanoparticles. The results achieved are remarkably encouraging and represent a significant advancement in the field of oncological nanomedicine. The complex biodistribution observed validates the importance of the MPS and other organs in mediating the interaction and processing of these nanoparticles. Moreover, the variability in therapeutic response among the animals, while highlighting the need for further studies, underscores the potential efficacy of ^198^AuNPs BSA in cancer treatment.

The primary accomplishment of this study is to demonstrate the significant therapeutic potential of the nanoparticles, with a focus on cases where there was complete regression of the tumor mass. The results are undoubtedly promising and signal an innovative path for therapeutic approaches against cancer. While recognizing that further investigations are needed, the data presented here suggest a research frontier that is not only promising from a scientific viewpoint but also has profound implications for improving public health and the general well-being of the population.

The hematological study in mice treated with BSA-coated gold nanoparticles showed that even after undergoing experimental treatment, most of the animals' hematological parameters remained close to or within the normal range. These results suggest that ^198^AuNPs BSA has a promising safety profile, at least with respect to the hematological system. When considering the potential application of these nanoparticles in oncological therapies, the current data is encouraging and highlights the need for further investigations. Ultimately, the research with ^198^AuNPs BSA stands out not only for its innovative merit but also for its therapeutic potential in the treatment of prostate cancer.

## Figures and Tables

**Table 1:** Concentrations and volumes (in µL) of AuNPs BSA used in cytotoxicity assays with LNCaP, PC-3, RWPE-1, and HUVEC cell lines.

NPsAu BSA Concentration	Volume in µL
1,0858x10^15^	20,0
9,5011x10^14^	17,5
8,1438x10^14^	15,0
6,7865x10^14^	12,5
5,4292x10^14^	10,0
4,0719x10^14^	7,5
2,7146x10^14^	5,0
1,3573x10^14^	2,5

**Table 2:** Therapeutic Efficacy of AuNPs BSA in BALB/c Nude mice with PC-3 prostate tumors. The blue section corresponds to Group 1 (G1) mice, while the yellow section refers to Group 2. The first column individually identifies each treated mouse, followed by the column specifying the administered radioactive activity. The following columns show the measurements of tumor volume at the beginning and after 22 days of treatment. The skull icon signifies the death of the mouse. The absence of a value in the mm³ column indicates that the tumor mass was so small that it could not be measured by the digital caliper.

ID Treated Animals	Injected Activity (µCi)	Initial Volume mm^3^	Final Volume mm^3^	Tumor Volume Variation
A1 9111	412,00	11,83	N	N
A2 9115	417,00	107,63	-	-100%
A3 9138	472,00	131,70	N	N
A4 9137	438,00	64,87	54,27	-16%
A5 9136	402,00	72,43	N	N
A6 9135	429,00	138,59	229,21	65%
				
A1 9113	762,00	18,31	N	N
A2 9134	640,00	25,10	8,98	-64%
A3 9133	414,00	54,67	66,56	22%
A4 9132	410,00	20,04	16,73	-17%
A5 9114	559,00	57,96	-	-100%
A6 9119	268,00	16,58	-	-100%

**Table 3:** Summary of the main parameters from the complete blood count of mice on the 22nd day after treatment, providing insights into the hematological effects of ^198^AuNPs BSA.

Laboratory Data: LAB & VET Veterinary Diagnosis and Consulting									
Av Escola Politécnica, 4445 - Rio Pequeno/SP - Brasil									
Veterinarian Responsible for the report: Naiadi A. Publio - CRMV-SP 32904									
Parameters Collected from the Complete Blood Count									
ID Animal	Erythrocytes (6,60 a 10,0 x 10^6^/µL)	Hemoglobin (14,3 a 17,3 g/dL)	Hematocrit (44% a 51%)	Observations Red Series	Leukocytes (4,40 a 8,30 x10^3^/µL)	Observations White Series	Platelet Count (282 a 481 x 10^3^/µL)	Hematozoan Research	µCi Dose Injected
9119	6,77 x 10^6^ / µL	12,48 g/dL	38%	NCM	4,74 x 10^3^ / µL	HN + - -	503 x 10^3^ / µL	AHL	198
9135	7,92 x 10^6^ / µL	16,31 g/dL	50%	NCM	112,40 x 10^3^ / µL	HN + + - TN + + - HN + - - PNDSN	243 x 10^3^ / µL	AHL	3,77
9115	6,55 x 10^6^ / µL	13,69 g/dL	42%	NCM	3,49 x 10^3^ / µL	NCM	252 x 10^3^ / µL	AHL	3,77
9133	5,26 x 10^6^ / µL	12,2 g/dL	37%	MAP	35,34 x 10^3^ / µL	HN + - -	581 x 10^3^ / µL	AHL	380
9137	7,94 x 10^6^ / µL	13,76 g/dL	42%	NCM	10,52 x 10^3^ / µL	HN + - -	542 x 10^3^ / µL	AHL	390
9114	5,21 x 10^6^ / µL	11,72 g/dL	36%	MAP	11,23 x 10^3^ / µL	HN + - -	203 x 10^3^ / µL	AHL	506
9134	6,55 x 10^6^ / µL	13,02 g/dL	40%	NCM	5,35 x 10^3^ / µL	NCM	694 x 10^3^ / µL	AHL	560

**Abbreviations:** NCM: Normal cell morphology, MAP: Mild anisocytosis and polychromasia, HN: Hypersegmented neutrophils, TN: Toxic neutrophils, PNDSN: Presence of neutrophils with a donut-shaped nucleus, AHL: Absence of hemoparasites in lamina
